# Unpacking the Mood States of Children and Youth in Saskatchewan, Canada, in the Context of the COVID-19 Pandemic: Insights from the “See Us, Hear Us 2.0” Study

**DOI:** 10.3390/children12010079

**Published:** 2025-01-10

**Authors:** Nahin Shakurun, Tamara Hinz, Daniel A. Adeyinka, Nazeem Muhajarine

**Affiliations:** 1 Department of Community Health and Epidemiology, College of Medicine, University of Saskatchewan, 107 Wiggins Road, Saskatoon, SK S7N 5E5, Canada; nahin.shakurun@usask.ca; 2 Saskatchewan Population Health and Evaluation Research Unit (SPHERU), University of Saskatchewan, 104 Clinic Place, Saskatoon, SK S7N 2Z4, Canada; 3 Department of Psychiatry, College of Medicine, University of Saskatchewan, 103 Hospital Drive, Saskatoon, SK S7N 0W8, Canada; tamara.hinz@usask.ca; 4 Saskatchewan Health Authority, Royal University Hospital, Saskatoon, SK S7N 0W8, Canada; daniel.adeyinka@saskhealthauthority.ca

**Keywords:** COVID-19, pandemic, post-COVID era, mood states, CRISIS scale, children and youth

## Abstract

Background/Objectives: The COVID-19 pandemic created a growing need for insights into the mental health of children and youth and their use of coping mechanisms during this period. We assessed mood symptoms and related factors among children and youth in Saskatchewan. We examined if coping abilities mediated the relationship between risk factors and mood states. Methods: “See Us, Hear Us 2.0”, a cross-sectional study of 563 child–parent dyads, provided the data. The dependent variable, current mood state, was measured by the CoRonavIruS health Impact Survey (CRISIS) scale. Independent variables included sociodemographics, behaviors, household conditions, and coping ability. Multiple linear regression and mediation analyses were conducted, ensuring sample representativeness with sampling weights and addressing missing data through multiple imputations. Results: The participants reported mood symptoms (“moderate” to “extreme”) ranging from 23% to 38% on the CRISIS scale. Factors such as older children, hybrid learning, disrupted activities, and increased screen time worsened moods. The ethnic minority groups (BIPOC) living in mid-sized cities/towns experienced more negative moods compared to Whites residing in cities. Coping ability mediated the relationship between extracurricular activities and mood states. Conclusions: Our results underscore the importance of tailored interventions, recognizing the diverse needs of specific age groups, gender identities, and ethnicities and addressing the adverse effects of the pandemic-related disruptions on the mental health and well-being of school children in Saskatchewan. Our study also suggests prioritizing the diverse needs of children and youth during the planning and implementation of mental health services in the province.

## 1. Introduction

In the post-COVID era, the mental health of children remains a crucial concern, as many are still struggling to cope with the psychological consequences of the pandemic. Stress and emotional instability have been intensified by disruptions in routine, prolonged isolation, and educational setbacks [1,2,3,4]. Although routines are returning to normal, some children still struggle to readjust to social settings and academic demands [5]. These challenges have left enduring effects on emotional and cognitive development, highlighting the importance of addressing mental health needs in this new landscape.

Several studies conducted during the pandemic revealed heightened mental health symptoms among children and youth [6,7]. A French study demonstrated that depression (29%), anxiety (49%), and irritability (51%) were more reported by 6- to 17-year-old children [8]. Hawke et al. [9] employed the CoRonavIruS health Impact Survey (CRISIS) scale (range: from 1 to 5) in their study, which specifically measures the symptoms of mental health challenges associated with COVID-19, and found a mean mood score of 3.14 (standard deviation = 0.77) for the 14–28-year-old community sample in Ontario, indicating a deterioration from the prepandemic level. A study of 8–18-year-old children and youth in Saskatchewan revealed that the point prevalence of medium to high anxiety and depression was 10.19% and 9.26%, respectively, in 2021 [10].

Early studies on COVID-19 found that mental health problems were more pronounced among older children and girls [11,12]. Mounting evidence suggests the link between urban–rural location and economic downturn to psychological well-being [10,13]. Similarly, a sudden shift to online or remote learning modalities exacerbated many psychological difficulties, e.g., anxiety, depression, inattention, loneliness, etc. [14,15,16,17]. Many studies have reported the impact of health behaviors (i.e., physical activity, sleep patterns, and screen use) on mental health symptoms [18,19,20,21]. A study of Iranian high school students revealed that moderate-to-vigorous physical activity was significantly associated with the negative moods such as anxiety, depression, and stress [22]. Another study revealed that more physical activity and less screen time were associated with better mental health for 6- to 17-year-old American children [23]. Additionally, economic downturn and interpersonal conflicts among family members potentially risk children to mental health adversities [24,25]. Longitudinal studies also reported that children and youth continue to suffer from psychological symptoms long after the COVID-19 pandemic [26,27]. The National Survey of Children’s Health (NSCH) reported that about one in five adolescents (12–17 years old) in the United States had a diagnosed mental or behavioral condition in 2023 along with disengagement from school and difficulty in making social connections [28].

According to the Canada 2021 census profile, nearly 28% of the population in Saskatchewan was under 19 years of age [29]. Saskatchewan’s unique population context warrants targeted analysis of children’s mental health in the post-COVID-19 era due to its distinct demographic and geographical characteristics. With 35% of the population living in rural areas, vast geographical distances with many communities located hours away from major urban centers, and 16% of the population being Indigenous, Saskatchewan faces singular challenges in mental health service delivery [30]. The resource-based economy, higher rates of child poverty in certain regions, and limited access to specialized mental health services outside urban centers create additional vulnerabilities [31,32]. These factors, combined with COVID-19′s disruption of social support systems and educational services and the government’s particular response, necessitate a focused investigation of different dimensions of children’s mental health along with potential modifiable factors that can provide the foundation for policies and interventions to strengthen provincial prevention measures.

The goal of this study was to report specific vulnerability factors of children and youth in Saskatchewan in the latter stages of the COVID-19 pandemic. To achieve this, our primary objective was to investigate different levels of mood symptoms present among children and youth at the end of the 2021–2022 academic year in Saskatchewan. The second objective was to provide evidence of associated factors with the mood states. We also analyzed the role of coping mechanisms as a potential mediator in the relationship between risk factors and negative mood states.

## 2. Materials and Methods

### 2.1. Study Design

We used data from “See Us, Hear Us (SUHU) 2.0”, a cross-sectional study that was conducted between 19 May and 21 July 2022. Data were collected through an online survey questionnaire. At the time of data collection, schools were reopened, and all public health restrictions (e.g., mask use, physical distancing, or other restrictions from the 2020–2021 school year) were lifted for the 2021–2022 school year [33]. Also, vaccination was continued for children aged 12 years and above.

### 2.2. Participants

This study employed a hybrid sampling design: an online community panel and a voluntary, self-selected sample from the general population. The Canadian Hub for Applied and Social Research-developed Saskatchewan online Community Panel recruits its members through rigorous and robust probability-based sampling methods, which ensures a more representative sample that closely aligns with the general demographic make-up of the province of Saskatchewan. The second source, a self-selected sample from the eligible population, was directed to the CHASR’s online survey. The invitation to participate in this study was sent by CHASR. To ensure the representativeness of the sample, weighting was applied using the iterative proportional method (also known as raking) based on age, gender, and location of residence using 2016 Canadian Census data for the targeted population [34].

A total of 563 child–parent dyads completed the online survey. In this study, the adult parent/caregiver of the child received the invitation to participate, not the child/youth directly. When there was more than one child/youth in the eligible age group present in the household (from 8 to 18 years), the child/youth whose birthday was closest to the date of survey was invited. This ensured one parent–child dyad per family or household participating in this study. These measures in place when recruiting would have had a countervailing effect to ‘rebalance’ any self-selection biases. Informed online consent was obtained before participation in the survey from both children and parents/caregivers. This study was approved by the Research Ethics Board of the University of Saskatchewan (Beh-2561).

### 2.3. Measures

#### 2.3.1. Dependent Variable

The outcome variable was current mood states, measured by the CoRonavIruS health Impact Survey Child Mood States Scale. The CRISIS scale measures the following 8 items: worry, self-reported depression, self-reported anxiety, attentiveness, fatigue, fidgetiness, loneliness, and irritability [35]. Amongst the other available mental health assessment tools, the CRISIS scale was selected as a newly validated and widely adopted measure of child mental health, specifically for the COVID-19 pandemic. Children and youth were asked to rate their moods on a 5-point Likert-type scale during the past two weeks preceding data collection (therefore referred to as “current mood states”). Items were recorded and averaged to generate a total score ranging from 0 to 4, with the high score indicating worse/negative moods. The categories of the items were collapsed to describe the prevalence of the components and reflect a mood symptom present at least moderately, compared to being “not at all” or “slightly”. Later, principal component analysis (PCA) was performed with the original items of the scale. The suitability of the items for data reduction was tested by Bartlett’s test of sphericity (*p*-value < 0.001), and the Kaiser–Meyer–Olkin (KMO) test for sampling adequacy exhibited a high adequacy level above 0.8 [36]. The principal component was retained with an eigenvalue >1, and that explained 100% of the variability of the CRISIS scale. The score for the principal component was generated and treated as a continuous outcome variable for further analysis. Throughout this paper, the CRISIS scale and the PCA score were interpreted in a manner where a lower score indicates a better mood and a higher score indicates a worse negative mood. The internal consistency of the measure was good for the current mental health of Canadian youth (Cronbach’s alpha = 0.88) [9].

#### 2.3.2. Independent Variables

Guided by the concept of Health Canada’s “Population Health Framework” (PHF) [37] and published articles, a set of independent variables was selected for this analysis. We considered learning modalities (in-person schooling, a mix of online and in-person, and online/homeschooling only) and any change in extracurricular activities as potential lifestyle factors. Any change in behavioral factors like physical activity, screen time, and sleep pattern in the past month before the data collection was assessed. Household financial stability and conflicts in the family were evaluated. We also considered the presence of positive COVID-19 cases at home, infection among acquaintances, and the coping capacity of the children and youth. Demographic information was collected for age, grade, gender, ethnicity, gross household income, location of residence, and parental immigration status. Details of these variables with their categorization and conceptual framework for this study are presented in the Supplementary Table (Figure S1 and Table S1).

### 2.4. Statistical Analysis

Data were analyzed using STATA statistical software version 17 [38]. Overall, the missing values ranged from 0% to 10%. We treated the “do not know or prefer not to answer” as missing for the income variable, so the missing values increased to 17%. Multiple imputation by chained equation technique was applied to address the missing data, and the pooled results were reported throughout this manuscript [39].

The sample characteristics were presented with frequencies and percentages. Bivariate analysis was performed between each independent variable and the outcome using simple linear regression. A *p*-value of <0.25 was used to select the candidate variables for multiple linear regression analysis. Multicollinearity among the selected variables was assessed by the mean variance inflation factor (VIF). Finally, the findings from the multivariable linear regression analysis with the forward selection approach were reported with unstandardized regression coefficients (b) and corresponding 95% confidence intervals (CI). A *p*-value of less than 0.05 was considered significant. Pre-selected interactions were assessed to determine the association of current mood states with age, gender, and ethnicity that changed across the location of residence, income levels, and immigration categories. Lastly, to address our third aim, the mediator role of children’s coping capacity was assessed between risk factors and the mood states [40]. The risk factors included in this study were selected based on published articles and on the advice from the child–parent advisory council and our clinical (child psychiatry) experience and insights.

## 3. Results

### 3.1. Sample Characteristics

Table 1 represents the demographic characteristics of our respondents. About 41.1% of the participants were 8–11 years old, 34.7% were 12–14 years old, and 24.1% were 15–18 years old. Over half of the respondents were in elementary grades (Grade 1–8) (63.4%). Approximately 48.0% of the children and youth self-identified as a boy, 47.4% as a girl, and 4.5% as other (neither boy nor girl). Regarding ethnocultural identity, the majority reported being White (79.6%), while one-fifth of the respondents identified as BIPOC (Black, Indigenous, and People of Color) (20.3%). Almost half of the respondents were from cities (Saskatoon/Regina) (49.9%). About 58.3% of respondents reported a yearly household income (pre-tax) of CAD 100,000 or higher, and about 15.5% of the respondents reported migrant status. Based on the census data of 2021, our sample is well representative of the targeted population of Saskatchewan [29].

### 3.2. Mood Symptoms and Associated Factors

**Figure 1:**
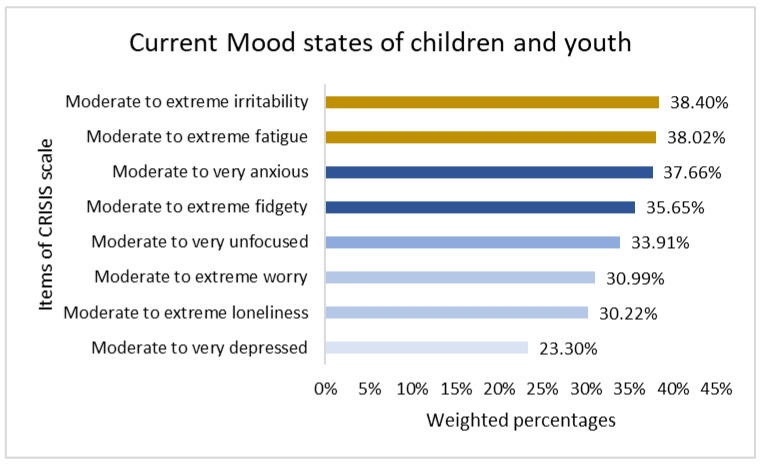
Prevalence of mood symptoms (from “moderate” to “extreme” in the CRISIS scale) in children and youth (8–18 years) in Saskatchewan.

In the 2021–2022 school year, children and youth reported varying levels of mood symptoms. Figure 1 illustrates the prevalence of eight mood symptoms as measured by the CRISIS scale. The prevalence of moderate to severe mood changes ranged from 23% to38%. The prevalence of moderate to extreme irritability (38.4%) and fatigue (38.0%) was higher, whereas the prevalence of moderate to severe depression was the lowest (23.3%). A detailed distribution of the different categories of the CRISIS items is presented in the Supplementary File (Table S2).

Table 2 shows the coefficients from univariate (unadjusted) and multiple regression models (adjusted). In the adjusted model, negative moods were significantly higher among 16–18-year-old children compared to 8–11-year-olds (b = 0.27; 95% CI: 0.02, 0.52; *p* < 0.05). Children who did not self-identify as boys/girls had 0.78 points higher mood scores than those who self-identified as boys (95% CI: 0.35, 1.21; *p* < 0.001). According to the location of residence, children residing in rural areas had significantly lower mood scores compared to those from the cities (Saskatoon/Regina) (b = −0.17; 95% CI: −0.34, 0.00; *p* < 0.05).

Among the factors related to daily life, hybrid learning modalities (b = 0.24; 95% CI: 0.04, 0.43; *p* < 0.05) and disrupted extracurricular activities (b = 0.18; 95% CI: 0.04, 0.33; *p* < 0.05) were significantly associated with higher mood scores in children and youth during the second academic year into the pandemic. Those who had “the same” amount of physical activities as earlier in the pandemic had scored more on the CRISIS scale compared to those who had increased activity (b = 0.21; 95% CI: 0.05, 0.37; *p* < 0.05). Likewise, increased screen time in the past month was significantly associated with increased mood score, 0.34 coefficient points higher, compared to those whose screen time had decreased (b = 0.34; 95% CI: 0.16, 0.53; *p* < 0.001). Additionally, conflicts among the family members were significantly associated with increased mood scores (a lot/somewhat more, compared to none/somewhat less; b = 0.32; 95% CI: 0.00, 0.64; *p* < 0.05). Children who said that they had less capacity to cope experienced significantly higher mood scores during this period (sometimes, b = 0.48; 95% CI: 0.34, 0.63; *p* < 0.001 and hardly ever, b = 0.70; 95% CI: 0.40, 0.99; *p* < 0.001).

**Figure 2:**
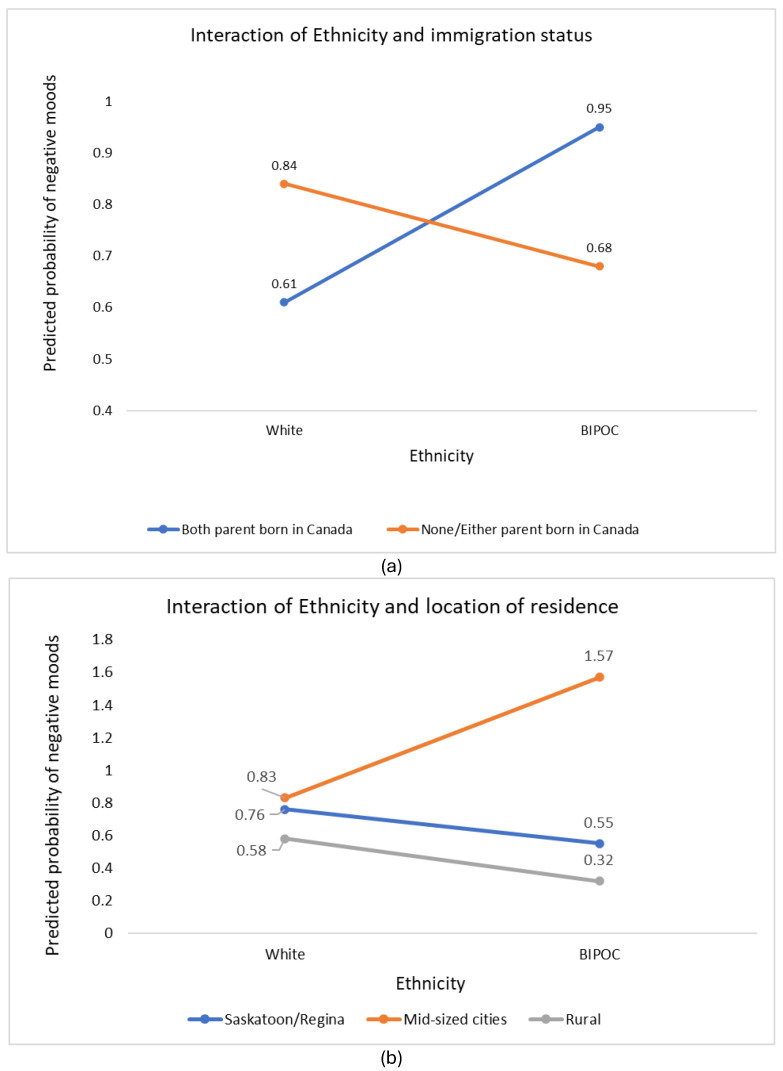
(**a**) Location of residence modifies the effect of ethnicity in predicting mood states in children and youth in Saskatchewan. (**b**) Immigration status modifies the effect of ethnicity in predicting negative mood states in children and youth in Saskatchewan.

We found a significant moderating effect involving ethnicity and location of residence. (Figure 2a). BIPOC children and youth were associated with lower mood scores, compared to white counterparts, for those who resided in urban centers or in rural communities. This association was significantly modified—in fact, reversed, or heightened—for those who lived in mid-sized communities. Another significant moderating effect involved ethnicity and immigration status. BIPOC children whose parents were Canadian-born were associated with a probability of higher mood scores compared to BIPOC children with at least one parent born outside of Canada (Figure 2b).

**Figure 3:**
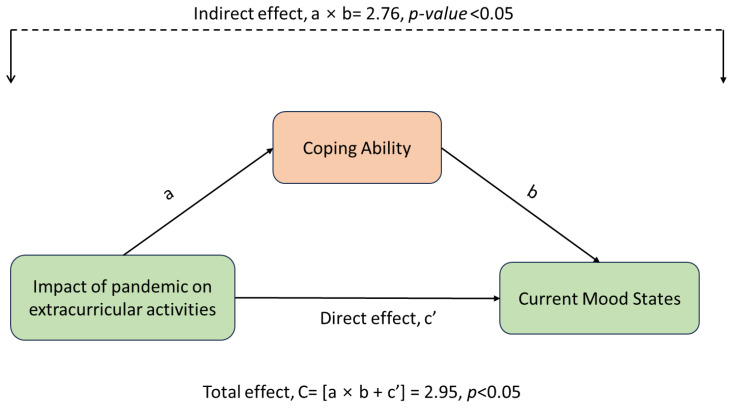
The mediational effect of coping ability of children and youth in the relationship between extracurricular activities and the current mood states of the respondents.

Figure 3 represents the mediational effect of coping ability. The effect of disrupted extracurricular activities on mood states of children and youth is significantly mediated through coping ability (indirect effect, a × b = 2.76; *p* < 0.05). The ratio of indirect effect to total effect (RIT = (a × b)/C) was 0.94, i.e., 94% of the total effect of disrupted extracurricular activities on the current mood states of children and youth in Saskatchewan was mediated by the respondent’s coping ability.

## 4. Discussion

The present study assessed the different levels of mood states and their correlates in children and youth using data from the “See Us, Hear Us 2.0” study in Saskatchewan, Canada. This study identified a significant mediational path to mood states through the coping ability of children and youth. We found that children and youth in Saskatchewan experienced varying levels of mood symptoms according to the CRISIS scale during the 2021–2022 academic year. More than one-third of the respondents had moderate to extreme irritability (38.40%) and fatigue (38.02%) among the eight components of mood symptoms, which are comparable with the previously published articles [41,42,43].

We found respondents aged 16 to 18 years reported higher mood scores, which is aligned with the findings from a study indicating increased emotional issues such as sadness and irritability among 9470 adolescents aged between 12 and 17 years old [44]. Conversely, during the first academic year (2020–2021), Saskatchewan children aged 8–11 experienced heightened anxiety and depression [10]. This shift from younger to older children may stem from biological, developmental (e.g., hormonal changes), and psychosocial factors (e.g., academic and interpersonal stresses) [45]. When comparing the mood scores across genders, there was an association between higher mood scores and respondents who did not identify themselves as boy or girl (such as those identifying as non-binary, two-spirit, or others). However, previous studies report results in the opposite direction. [25,44]. An Australian research emphasized increased depressive and anxiety symptoms in girls [46]. However, this disparity compels us to prioritize equity-deserving groups like sexual and gender minorities and underscore the necessity for tailored mental health support for them.

Similar to other studies, our research indicates that residential location has an impact on mental health [47,48]. We found an association between rural children and better mood states, compared to those in cities (Saskatoon/Regina) and mid-sized towns. It is possible that this reflects to a degree the relatively greater disruption of normal life and routine in cities compared to rural communities during the pandemic. Greater access to nature, lower-density population centers and events and reduced environmental stressors may have also contributed to the relative diminishment of poor mental health symptoms in rural children and youth. Additionally, strong community ties and support in rural areas may have aided in overcoming mental health challenges. However, further exploration with detailed qualitative components is required to understand the extent to which any social stigma or cultural norms may have hindered children and youth in rural areas reporting or accessing mental health services.

In addition to sociodemographic factors, this study also highlighted the diverse effects of the pandemic on lifestyle and behavioral factors. Our study participants who had hybrid learning settings reported higher mood scores compared to those who attended school in person. Prior research has reported the detrimental effects of online learning on mental health early in the pandemic [15,46]. Our findings emphasize that even after public health restrictions were lifted, children in hybrid learning during the second academic year in the pandemic struggled to return to normalcy. The current study examined the link between extracurricular activities and mood states among children and youth in Saskatchewan and found that respondents with disrupted extracurricular activities since the onset of the pandemic had higher mood scores. This aligns with the findings from prior studies carried out among adolescents in various Canadian provinces [49,50]. Oberle et al. [49] revealed that extracurricular activities are linked to increased life satisfaction and decreased anxiety and depression. Likewise, LaForge-MacKenzie et al. [50] found that extracurricular activities improved mental health outcomes for Ontario’s children and youth before and during the pandemic. We also identified that elevated screen time was responsible for increased mood scores, as observed in earlier pandemic studies [44,51,52]. Limited outdoor options during the pandemic’s onset exacerbated screen dependency.

About 33.18% of respondents experienced increased family conflicts during the second year of the pandemic, significantly linked to increased mood symptoms, consistent with SUHU 1.0 findings [10] and Australian research [46]. The latter observed more depressive symptoms in children with heightened conflicts with their fathers, whereas conflicts with siblings, friends, or mothers did not impact anxiety or depressive symptoms [46]. Family dynamics, particularly parent–child relationships, crucially influence children’s psychological well-being [53,54], especially during the pandemic when there was limited peer support available. Dysfunctional parenting processes, parental work–life conflicts, caregivers’ mental health, and financial strain exacerbated familial chaos or conflicts during COVID-19 [54,55,56,57].

In this research, we found that children and youth who had less ability to cope expressed higher mood scores. Vallejo-Slocker et al. [58] demonstrated that nonactive coping predicted worse mental health, whereas problem-solving served as a protective factor for Spanish children. Zhang et al. [57] showed positive coping style, such as positive appraisal and thinking, distancing, problem-solving, and help-seeking, was protective against depression, anxiety, and trauma-related distress. These findings were generally in line with our results. The assessment of the various coping strategies adopted by the respondents was beyond the scope of this study. Further detailed evaluation of coping methods adopted by the children and youth is required, preferably through qualitative investigations.

This study revealed significant interactions between ethnocultural identity, location of residence, and parental immigrant status in Saskatchewan. BIPOC children in mid-sized cities exhibited notably higher mood scores compared to their counterparts in cities (Saskatoon/Regina). Moreover, there is an association between BIPOC children and youth whose parents were Canadian-born and significantly higher mood scores compared to those whose parents were born outside of Canada. Our findings provided insights into the unique challenges faced by this group, highlighting persistent disparities among socially disadvantaged groups. During the first year of the pandemic, BIPOC children from low-income families were more likely to report low-to-moderate quality of life compared to higher-income counterparts [10]. These findings emphasize the necessity for specialized mental health services tailored to marginalized populations in Saskatchewan.

The mediational effect of coping ability was a unique finding of this study. Our findings identified a significant mediational path between extracurricular activities and mood states among children and youth with significant indirect and total effects through their coping ability. The potential connection between disrupted extracurricular activities and negative mood experiences as a coping mechanism in children and youth remains underexplored in the literature. Few studies have specifically examined the relationship between participation in extracurricular activities and the development of practical coping abilities in children [59,60]. Creative activities, such as music and art, keeping social connections, routing building and engaging in physical activities were proven as effective coping strategies for emotional wellbeing and fostering resilience during the pandemic [31]. A longitudinal study of 1162 Australian children demonstrated that participation in extracurricular activities predicted improved coping efficacy [61]. Effective coping skills have been associated with various positive outcomes for adolescents, including higher self-esteem, reduced symptoms of depression and conduct problems, and enhanced social and academic competence [59]. Further qualitative studies are essential for a more comprehensive understanding of the coping strategies adopted by the children and youth in the post-pandemic period.

We incorporated the eight components of mood by the principal component analysis, which allowed us to reduce the dimensionality of the data but retain maximum variability of the components. Missing data were addressed using appropriate techniques, which made our findings more informative and valid. Another strength of this study was to elucidate the mediating effect of coping ability on the relationship between extracurricular activities and mood state among Saskatchewan’s children and youth. As in any study, however, there are several limitations that should be noted. The cross-sectional survey data precluded revealing causal relationships. Although future longitudinal studies may tease out causality, cross-sectional studies have a place. They could reveal understanding of a problem, such as mental health, in real time and especially during an unprecedented pandemic. Due to multiple imputation methods, we were not able to test the separate path coefficients and establish the standardized mediational effect. Additionally, public health restrictions varied from province to province. Therefore, our results may not be generalizable in other jurisdictions.

## 5. Conclusions

Our study provides significant evidence on the magnitude of mood states of the children and youth, along with the associated factors, at the end of the second academic year since the pandemic began. The time when the data were collected, all the public health mandates were lifted, and schools resumed in-person classes. However, our study confirms the multidimensional mood experiences of children and youth in Saskatchewan at the end of the second academic year since the pandemic began. Our study also emphasized the significant sociodemographic and lifestyle factors that can inform policy changes and the reallocation of mental health resources to the equity-seeking groups while eliminating mental health disparities. Also, future research with qualitative components is warranted to understand the coping mechanism employed by the children and youth during this devastating situation. The collaboration between the researchers, mental health care providers, and policymakers is essential to design and implement targeted interventions for children and youth.

## Figures and Tables

**Table 1:** Demographic characteristics of the participants (n = 563); “See Us, Hear Us 2.0” study, Saskatchewan, Canada.

Characteristics	Frequency (%)	Imputed and Weighted Frequency (%)
Age		
8–11 years	246 (43.69)	232 (41.19)
12–15 years	198 (35.17)	195 (34.70)
16–18 years	119 (21.14)	136 (24.11)
Missing	0	
Grade		
Elementary	355 (63.06)	357 (63.47)
High	180 (31.97)	206 (36.53)
Missing	28 (4.97)	
Gender		
Boy	286 (50.80)	271 (48.06)
Girl	247 (43.87)	267 (47.43)
Others	25 (4.44)	25 (4.51)
Missing	5 (0.89)	
Ethnicity		
White	441 (78.33)	449 (79.69)
BIPOC	117 (20.78)	114 (20.31)
Missing	5 (0.89)	
Gross household income		
Less than CAD 100,000	197 (34.99)	235 (41.67)
CAD 100,000 or more	268 (47.60)	328 (58.33)
Missing	98 (17.41)	
Location of residence		
Saskatoon/Regina	265 (47.07)	281 (49.95)
Mid-sized city/town	49 (8.70)	88 (15.55)
Rural	192 (34.10)	194 (34.50)
Missing	57 (10.12)	
Parent immigration status		
Both born in Canada	461 (81.88)	476 (84.48)
Neither/at least one parent born in Canada	94 (16.70)	87 (15.52)
Missing	8 (1.42)	

**Table 2:** Unadjusted and adjusted regression models for factors associated with the mood states score of children and youth in Saskatchewan, Canada (results are presented as unstandardized coefficients with 95% confidence intervals).

Characteristics	Unadjusted Coefficient (95% CI)	Adjusted Coefficient (95% CI)
Age		
8–11 years	Ref	Ref
12–15 years	0.23 (0.04, 0.42) *	0.11 (−0.05, 0.26)
16–18 years	0.43 (0.21, 0.66) **	0.27 (0.02, 0.52) *
Grade		
Elementary	Ref	Ref
High	0.23 (0.04, 0.41) *	−0.01 (−0.21, 0.19)
Gender		
Boy	Ref	Ref
Girl	0.13 (−0.04, 0.31)	0.10 (−0.03, 0.23)
Others	0.93 (0.45, 1.40) **	0.78 (0.35, 1.21) **
Ethnicity		
White	Ref	Ref
BIPOC	0.06 (−0.17, 0.29)	0.05 (−0.27, 0.37)
Gross household income		
Less than CAD 100,000	Ref	Ref
CAD 100,000 or more	−0.25 (−0.43, −0.07) *	−0.07 (−0.24, 0.10)
Location of residence		
Saskatoon/Regina	Ref	Ref
Mid-sized city/town	0.36 (0.06, 0.66) *	0.07 (−0.18, 0.32)
Rural	−0.18 (−0.36, −0.01) *	−0.17 (−0.34, −0.00) *
Parent immigration status		
Both born in Canada	Ref	Ref
Neither/at least one parent born in Canada	−0.11 (−0.33, 0.11)	0.24 (−0.07, 0.55)
Learning method		
Attended in-person all the year	Ref	Ref
Mix of online and in-class learning	0.43 (0.18, 0.68) *	0.24 (0.04, 0.43) *
Online/Others	0.31 (−0.15, 0.76)	0.09 (−0.23, 0.41)
Impact of pandemic on extracurricular activities		
No impact	Ref	Ref
A little or a lot of impact	0.39 (0.22, 0.57) **	0.18 (0.04, 0.33) *
Change in physical activity		
More active	Ref	Ref
The same	0.28 (0.06, 0.50) *	0.21 (0.05, 0.37) *
Less active	0.47 (0.22, 0.71) **	0.15 (−0.08, 0.38)
Change in sleep pattern		
Better	Ref	Ref
The same	−0.23 (−0.41, −0.05) *	−0.10 (−0.25, 0.05)
Worse	0.52 (0.17, 0.87) *	0.21 (−0.10, 0.52)
Change in screen time		
Decreased	Ref	Ref
The same	0.16 (−0.02, 0.34)	0.07 (−0.09, 0.23)
Increased	0.66 (0.43, 0.89) **	0.34 (0.16, 0.53) **
COVID-19/COVID-like cases at home		
No cases	Ref	Ref
Positive case was present	0.29 (0.11, 0.48) *	0.20 (0.04, 0.36) *
Severity outside household		
No	Ref	Ref
Yes, severely ill/died	0.22 (−0.02, 0.46)	0.10 (−0.09, 0.28)
Financial stability		
Secure	Ref	Ref
Insecure	0.29 (0.10, 0.48) *	0.05 (−0.10, 0.21)
Family conflict		
None/somewhat less	Ref	Ref
No real change	−0.05 (−0.38, 0.29)	−0.07 (−0.38, 0.24)
Varied	0.24 (−0.11, 0.58)	0.06 (−0.26, 0.38)
A lot/somewhat more	0.59 (0.26, 0.92) **	0.32 (0.00, 0.64) *
Coping ability		
Most times/always	Ref	Ref
Sometimes	0.65 (0.50, 0.81) **	0.48 (0.34, 0.63) **
Hardly ever	0.97 (0.60, 1.34) **	0.70 (0.40, 0.99) **

N.B. * Significance at *p* < 0.05; ** Significance at *p* < 0.001.

## Data Availability

The data presented in this study are available upon reasonable request.

## Supplementary Materials

The following supporting information can be downloaded at: https://www.mdpi.com/article/10.3390/children12010079/s1. Figure S1: Conceptual framework adapted from Population Health Framework (Diagram courtesy: Dr. William Pickett); Table S1: Independent variables and their categorization; Table S2: Distribution of mood states with missing values (N = 563).
