# Supplementary material for: Unpacking the Mood States of Children and Youth in Saskatchewan, Canada, in the Context of the COVID-19 Pandemic: Insights from the “See Us, Hear Us 2.0” Study
Source: Children (Basel). 2025 Jan 10;12(1):79. doi: 10.3390/children12010079 (PMC11763707; doi:10.3390/children12010079)
Supplement: Supplementary file 1 [file children-12-00079-s001.zip › children-3340283-supplementary.pdf]

Title: Unpacking the mood states of children and youth in Saskatchewan, Canada in the context of the COVID-19 pandemic: Insights from See Us, Hear Us 2.0 Study

Supplementary file

Figure S1 Conceptual framework adapted from Population Health Framework (Diagram courtesy: Dr. William Pickett)

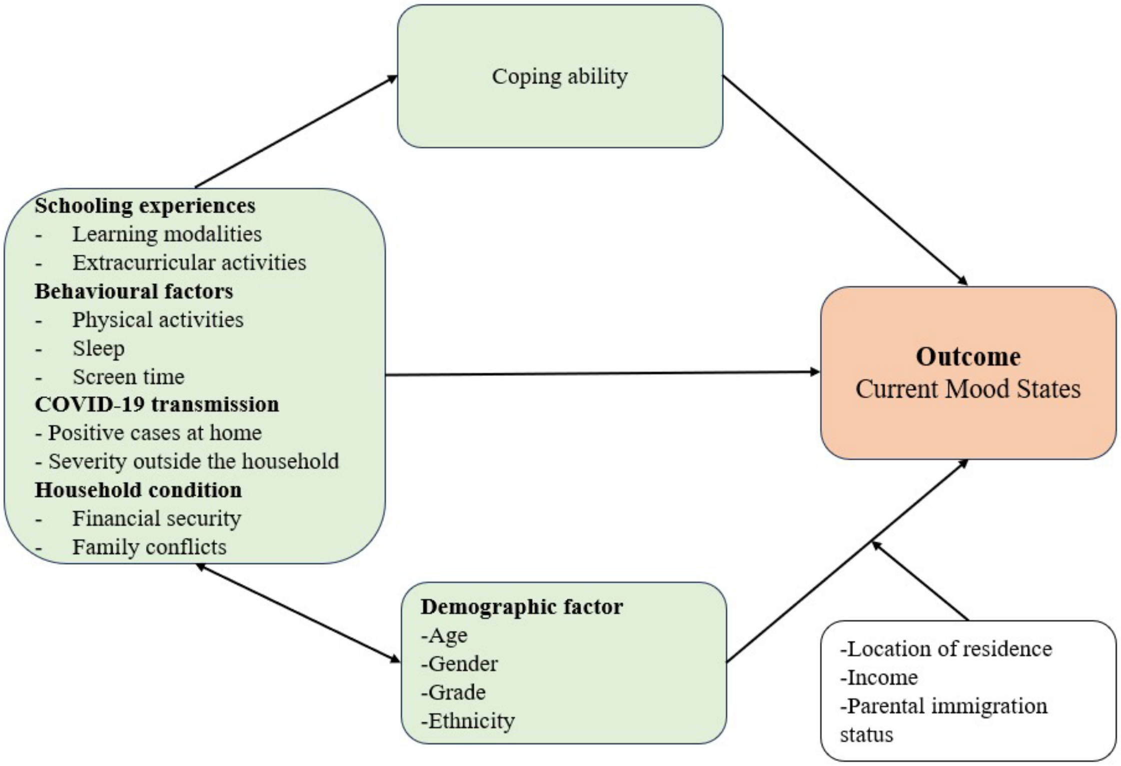

Table S1 Independent variables and their categorization

Variable	Description	Categorization
Age	Age of the respondents (Children) in years	0. 8-11 years<br>1. 12-15 years<br>2. 16-18 years
Grade	Educational level of the child	0. Elementary<br>1. High
Gender	Gender of the child	0. Boy<br>1. Girl<br>2. Others (non-binary, two-spirit, and others)
Ethnicity	Ethnic identity of the child	0. White<br>1. BIPOC

Gross household income	Total household income (before tax)	2. Less than $100,000<br>3. $100,000 or more
Location of residence	Place of residence	0. Saskatoon/Regina<br>1. Mid-sized city/town<br>2. Rural
Parent immigration status	Immigration status of the parents	0. Both born in Canada<br>1. Neither/at least one parent born in Canada
Learning method	Mode of learning in the 2021-2022 academic year	0. Attended in-person all the year<br>1. Mix of online and in-class learning<br>2. Online/ Others
Impact of pandemic on extracurricular activities	Any impact of pandemic on extracurricular activities	0. No impact<br>1. A little or a lot of impact
Change in physical activity	Change in the physical activity in past month prior to the survey	0. More active<br>1. The same<br>2. Less active
Change in sleep pattern	Change in the sleep pattern in past month prior to the survey	0. Better<br>1. The same<br>2. Worse
Change in screen time	Change in the screen time in past month prior to the survey	0. Decreased<br>1. The same<br>2. Increase
COVID-19/COVID-like cases at home	COVID-positive cases at home	0. No cases<br>1. Positive case was present
Severity outside household	If acquaintances outside the household who are close to the child/youth were ill or died from COVID	0. No<br>1. Yes, severely ill/died
Financial stability	Household's level of financial stability	0. Secure<br>1. Insecure
Family conflict	Conflict inside the household since the pandemic began	0. None/somewhat less<br>1. No real change<br>2. A lot/ somewhat more
Coping ability	The ability of children to cope when they were upset/unhappy	0. Most times/ always<br>1. Sometimes<br>2. Hardly ever

Table S2 Distribution of mood states with missing values (N=563)

Mood states	**Frequency (%)**	**Missing n (%)**
**Worry**		4 (0.71)

Not worried at all	208 (36.94)	
Slightly worried	183 (32.50)	
Moderately worried	116 (20.60)	
Very worried	38 (6.75)	
Extremely worried	14 (2.49)	
**Self-reported depression**		4 (0.71)
Very happy/cheerful	78 (13.85)	
Moderately happy/cheerful	176 (31.26)	
Neutral	187 (33.21)	
Moderately depressed/sad/unhappy	101 (17.94)	
Very depressed/sad/unhappy	17 (3.02)	
**Self-reported anxiety**		4 (0.71)
Very relaxed/calm	67 (11.90)	
Moderately relaxed/calm	134 (23.80)	
Neutral	161 (28.60)	
Moderately anxious/nervous	165 (29.31)	
Very anxious/nervous	32 (5.68)	
**Poor concentration**		5 (0.89)
Very focused/attentive	80 (14.21)	
Moderately focused/attentive	155 (27.53)	
Neutral	145 (25.75)	
Moderately unfocused/ distracted	133 (23.62)	
Very unfocused/ distracted	45 (7.99)	
**Fidgety**		5 (0.89)
Not fidgety/restless at all	149 (26.47)	
Slightly fidgety/restless	207 (36.77)	
Moderately fidgety/restless	126 (22.38)	
Very fidgety/restless	49 (8.70)	
Extremely fidgety/restless	27 (4.80)	
**Fatigue**		5 (0.89)

Not fatigue/tired at all	119 (21.14)	
Slightly fatigue /tired	231 (41.03)	
Moderately fatigue /tired	119 (21.14)	
Very fatigue /tired	65 (11.55)	
Extremely fatigue /tired	24 (4.26)	
**Irritability**		6 (1.07)
Not irritable/easily angered at all	153 (27.18)	
Slightly irritable/easily angered	189 (33.57)	
Moderately irritable/easily angered	143 (25.40)	
Very irritable/easily angered	47 (8.35)	
Extremely irritable/easily angered	25 (4.44)	
**Loneliness**		5 (0.89)
Not lonely at all	228 (40.50)	
Slightly lonely	173 (30.73)	
Moderately lonely	92 (16.34)	
Very lonely	50 (8.88)	
Extremely lonely	15 (2.66)	
